# Differential Biotransformation of Glycyrrhizin by Licorice-Derived Endophytic Fungi and Accumulation-Promoting Effects of Fungal Inoculation

**DOI:** 10.3390/ijms27125444

**Published:** 2026-06-16

**Authors:** Xin Zuo, Guangxi Ren, Dan Jiang, Chunsheng Liu

**Affiliations:** School of Chinese Materia Medica, Beijing University of Chinese Medicine, Beijing 102488, China

**Keywords:** licorice, endophytic fungi, *β*-glucuronidase, glycyrrhizin, biotransformation, endophyte–host interaction

## Abstract

Medicine food homology (MFH) plants are rich in nutrients and bioactive specialized metabolites, and their endophytic fungi mediate key biotransformation of host secondary metabolites. Licorice, a representative MFH herb, accumulates glycyrrhizin (GL) as its dominant bioactive triterpenoid saponin. Its hydrolysates glycyrrhetinic acid (GA) and glycyrrhetinic acid 3-*O*-mono-*β*-D-glucuronide (GAMG) show stronger bioactivity and bioavailability than GL. However, the enzymatic mechanisms of licorice-derived endophytic fungi-mediated GL biotransformation remain unclear. Here, nine licorice endophytic fungi were screened for significant GL-inducible *β*-glucuronidase activity. Four functional GH2 *β*-glucuronidase were obtained by prokaryotic and eukaryotic expression systems, and confirmed to catalyze glycyrrhizin biotransformation via two distinct hydrolytic pathways. Inoculation of these four strains into licorice markedly enhanced host glycyrrhizin accumulation. This study provides novel enzymatic resources for the efficient bioproduction of high-value glycyrrhizin derivatives, and proposes a green strategy to improve glycyrrhizin content in licorice, deepening the understanding of endophyte–host metabolic crosstalk in medicinal herbs.

## 1. Introduction

Medicine food homology (MFH) plants possess both nutritional value for promoting health and therapeutic efficacy in alleviating various diseases, attracting increasing and widespread attention from researchers [1]. Licorice, as a classic MFH plant, has a long history of medicinal use in China and is also widely employed in the food and health industry [2,3]. To date, more than 400 secondary metabolites have been isolated from licorice, mainly including about 300 flavonoids and 60 triterpene saponins [4]. Pharmacological studies indicate that flavonoids (such as liquiritin) and triterpenoids (such as glycyrrhizin), serving as quality markers for licorice, are key constituents responsible for its pharmacological activities including anti-inflammatory, antiviral, antibacterial, immunomodulatory, and antioxidant effects [3,5]. Glycyrrhizin is a triterpene saponin unique to licorice root, exhibiting significant pharmacological activity including anti-inflammatory, antibacterial, antiviral, and anticancer effects [6]. Human pharmacokinetic studies further indicate that the hydrolysis products resulting from the action of gut bacteria on glycyrrhizin are the forms responsible for its pharmacological activity within the human body [7]. Among these, glycyrrhetinic acid (GA) and glycyrrhetinic acid 3-*O*-mono-*β*-D-glucuronide (GAMG), as hydrolysis products of glycyrrhizin, typically exhibit superior bioactivity and bioavailability [6,8]. Furthermore, triterpenoids such as glycyrrhizin are known to act as both defensive phytochemicals and signaling molecules in plant–microbe communication [9,10,11,12].

Endophytic fungi are integral members of the plant holobiont, contributing to host growth, stress resilience, and biosynthesis of bioactive compounds [13]. These fungi often possess strong secretion systems and diverse enzyme repertoires, including oxidoreductases, hydrolases, and laccases, enabling them to degrade complex organic molecules. Notably, endophytes can express “detoxifying enzymes,” such as *β*-glucuronidases, that help them metabolize host-derived phytochemicals and adapt to the chemical environment of their hosts, thereby maintaining a stable symbiotic relationship [14]. *β*-glucuronidases (EC 3.2.1.31, GUS) are glycosyl hydrolases that catalyze the cleavage of glucuronide glycosidic bonds, releasing the aglycone from its conjugated form [15]. Most *β*-glucuronidases implicated in glycyrrhizin hydrolysis belong to glycoside hydrolase family 2 (GH2), as documented in the Carbohydrate-Active enZYmes (CAZy) database [16]. Several microbial GUS enzymes capable of transforming glycyrrhizin have been characterized, including enzymes from *Staphylococcus pasteuri* [17], *Aspergillus terreus* Li-20 [18], *Chaetomium globosum*, and *Talaromyces pinophilus* [19,20]. Our previous work also identified two *β*-glucuronidases (Z6GH2 and Z15GH2) from licorice endophytes that catalyze glycyrrhizin hydrolysis through different mechanisms [21]. These findings underscore the ecological and biochemical importance of microbial GUS enzymes in saponin metabolism and highlight their potential for producing high-value derivatives such as GAMG and GA.

To better understand the molecular basis of glycyrrhizin biotransformation by licorice endophytes, we systematically investigated their metabolic activity and *β*-glucuronidase expression profiles. Using comparative and functional genomics, we screened for GH2 candidate genes potentially involved in glycyrrhizin hydrolysis. Functional validation was performed via prokaryotic and eukaryotic expression systems. This study aims to elucidate the enzymatic mechanisms by which endophytic fungi metabolize glycyrrhizin, laying the groundwork for further obtaining bioactive compounds with higher activity and bioavailability.

## 2. Results

### 2.1. Glycyrrhizin-Metabolizing Activity and β-Glucuronidase Expression in Licorice Endophytic Fungi

To investigate the glycyrrhizin-metabolizing potential of licorice endophytic fungi, 33 isolates were screened on minimal medium with glucose (MM_glu) or glycyrrhizin (MM_GL) as the sole carbon source. A total of 18 strains, including H3, Z9, Z11, Z25, H24, Z32, H5, H6, H7, Z17, Z20, Z22, Z35, H1, H16, H11, I13 and I14, showed robust growth on MM_GL plates (Figure S1A), while the remaining 15 strains exhibited limited or no growth (Figure S2A). Subsequent HPLC analysis after glycyrrhizin fermentation revealed that 16 of the 18 strains (excluding Z17 and Z20) actively metabolized glycyrrhizin, with conversion rates ranging from 29.46~100% (Figure S1B,C). This result indicated that 16 of the 33 endophytic fungal to be tested possessed the ability to metabolize glycyrrhizin.

**Figure 1:**
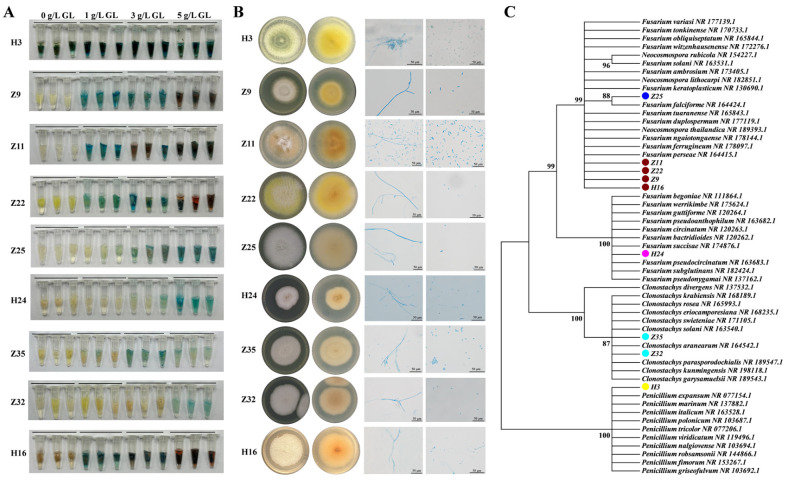
Identification of *β*-glucuronidase activity and the characterization of the licorice endophytic fungi. (**A**) GUS staining of fungal mycelia co-cultured with glycyrrhizin. (**B**) Growth morphology and microscopic structure of selected strains on PDA medium. (**C**) Phylogenetic trees based on ITS sequences. The values indicate the confidence level of each branch.

To explore whether *β*-glucuronidase (GUS) is involved in glycyrrhizin metabolism, GUS staining was conducted following co-culture with glycyrrhizin (Figure S2B). Strain H3 exhibited strong GUS activity under all glycyrrhizin concentrations tested (1, 3, and 5 g/L), indicating constitutive expression of *β*-glucuronidase (Figure 1A). In contrast, strains Z9, Z11, Z22, Z25, Z32, Z35, H24, and H16 showed GUS staining only after glycyrrhizin treatment, suggesting glycyrrhizin-inducible expression (Figure 1A). Among them, the *β*-glucuronidase activity was expressed in Z9, Z22, and H16 after co-culture with 3 and 5 g/L glycyrrhizin; Z11 and Z35 showed *β*-glucuronidase activity only after co-cultivation with 1 g/L or 3 g/L glycyrrhizin, but H24 and Z32 showed *β*-glucuronidase activity only at a high (5 g/L) glycyrrhizin concentration (Figure 1A). The remaining seven tested strains showed no detectable *β*-glucuronidase activity under any condition. These results indicate that all strains except H3 exhibit glycyrrhizin concentration-dependent *β*-glucuronidase expression. We therefore propose that licorice endophytic fungi may initiate glycyrrhizin biotransformation via glycyrrhizin-induced expression of *β*-glucuronidase.

Nine strains with both glycyrrhizin-metabolizing activity and GUS expression were further characterized morphologically and phylogenetically. H3 displayed typical *Penicillum* morphology, with white flocculent mycelium, broom-like conidiophores and smooth spherical conidia (Figure 1B). Z9, Z11, Z22, Z25, H16 and H24 had the typical characteristics of *Fusarium*: its mycelium was cotton-wool-like with different colors, the conidiophore was elongated and obviously branched, and the conidia were mostly separated and the ends of the spores were slightly curved and had a sickle shape. Z32 and Z35 were morphologically characterized by the genus *Clonostachys*, which had white flocculent mycelium with discoidal growth and ellipsoid or ovoid spores. A phylogenetic tree was further constructed based on ITS sequences from the nine licorice endophytic fungi and their closely related species, with bootstrap values below 75% removed, revealing the evolutionary relationships among the strains (Figure 1C). Of these, H3 was more closely related to *P. expansum*; Z32 and Z35 were classified in different species branches of the *Clonostachys*. Additionally, six endophytic fungi showed a high homology with *Fusarium* and have been classified into two different subclusters, with Z25, Z11, Z22, Z9, and H16 belonging to the same subcluster, and H24 belonging to the other subcluster. The assembly results indicate that the genome sequencing of the nine endophytic fungal strains was reliable and accurate (Table S2). Further analysis based on the NR annotation results revealed that the species annotation for H3 was consistent with the ITS identification results (Figure S3). Both Z35 and Z32 were annotated as *C. rhizophaga*, whilst the five endophytic fungi belonging to the *Fusarium* were annotated as two different species, *F. vanettenii* (Z9, Z11, Z22 and H16) and *F. falciforme* (Z25). In contrast to the ITS identification results, all 7744 coding genes of H24 were annotated to *C. rosea*. These results suggest that different endophytic fungi retained a certain degree of interspecific conservatism during the evolutionary process, and also evolved different intraspecific variability.

### 2.2. Candidate β-Glucuronidases Exhibit Potential Glycyrrhizin-Binding Activity

**Figure 2:**
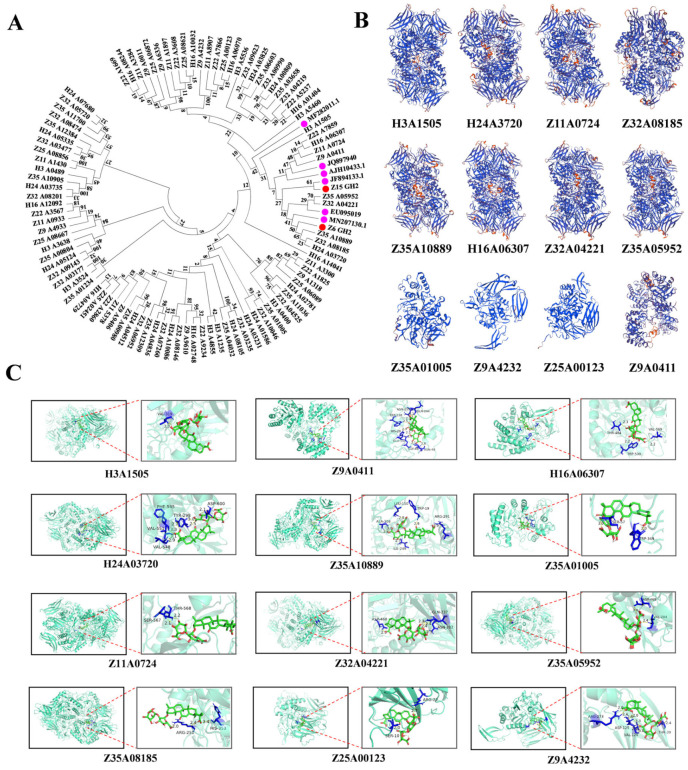
Screening and structural analysis of candidate GH2 proteins. (**A**) Phylogenetic analysis of candidate GH2 proteins from licorice endophytic fungi and known *β*-glucuronidases. (**B**) Predicted 3D structures of selected GH2 proteins based on homology modeling. (**C**) Molecular docking models showing the interaction between candidate GH2 protein and glycyrrhizin ligands.

Genome-wide CAZy annotation of the nine glycyrrhizin-metabolizing endophytic fungi revealed that glycoside hydrolases (GHs) were the most abundant class of carbohydrate-active enzymes (Figure S4). From these, 93 genes encoding putative GH2 family *β*-glucuronidases were identified across strains H3, Z9, Z11, Z22, Z25, Z32, Z35, H24, and H16. Phylogenetic analysis identified 10 candidate GH2 proteins clustered in the same branch with reported *β*-glucuronidases with glycyrrhizin catalytic activity. Among them, Z32A04221 and Z35A05952 were more closely related to the *β*-glucuronidase Pgus (EU095019) from *A. oryzae*. Z22A7859, H16A06307, Z11A0724 and Z9A0411 were grouped to the *β*-glucuronidase from *E. coli* (AJH10433) and *A. ustus* Li-62 (JQ897940). Meanwhile, Z35A10889, Z32A08185 and H24A03720 had closer affinities with *β*-glucuronidase from *Chaetomium globosum* (MN207130) and the licorice endophytic fungus *A. terreus* (Z6GH2) (Figure 2A). Therefore, these GH2 proteins, which exhibited higher sequence similarity to known functional *β*-glucuronidases, were selected as candidate enzymes potentially involved in the microbial transformation of glycyrrhizin.

Motif analysis using MEME further supported their functional relevance (Figure S5). H3A1505, Z32A04221, Z32A08185, Z35A05952, and Z11A0724 exhibited all 10 conserved GH2 catalytic motifs shared with reported glycyrrhizin-hydrolyzing enzymes (JQ897940, EU095019, JF894133, MF282011, MN207130, and AJH10433). Z35A10889 and H16A06307 displayed nine conserved motifs, while H24A03720 retained seven. These candidate GH2 proteins with conserved functional motifs were also identified as candidate proteins that may have similar catalytic functions for glycyrrhizin. Taking into account the sequence length, distribution characteristics, and identity annotation results of GH2 proteins, Z35A01005 (72.2%), Z9A4232 (89.7%), and Z25A00123 (98.9%) were subsequently identified as candidate proteins.

Predicted 3D structures of the 12 candidate GH2 proteins exhibited high confidence, with EMQE scores ranging from 0.81 (Z9A0411)~0.96 (Z9A4232) (Table S3), and similarities ranging from 59.85% (Z35A10889)~94.53% (Z9A4232), indicating that the predicted protein models have a high confidence (Table S3). In particular, H3A1505, H24A3720, Z11A0724, Z32A08185, H16A06307, Z32A04221, and Z35A05952 also had similar tetrameric conformations (Figure 2B). AutoDock simulations further demonstrated favorable binding between glycyrrhizin and all 12 candidate GH2 proteins, with predicted binding free energies ranging from −3.45 (Z32A04221) to −7.05 (Z35A05952) kcal/mol, suggesting potential ligand–enzyme interactions (Table S4). Pymol visualization confirmed that the glucuronic acid residues of glycyrrhizin were capable of forming hydrogen bonds with catalytic residues located within the active sites of these proteins (Figure 2C), with distances below 3.3 Å. Collectively, these results support the hypothesis that these GH2 candidates possess potential catalytic activity toward glycyrrhizin.

### 2.3. β-Glucuronidase from Licorice Endophytic Fungi with Glycyrrhizin Catalytic Activity

**Figure 3:**
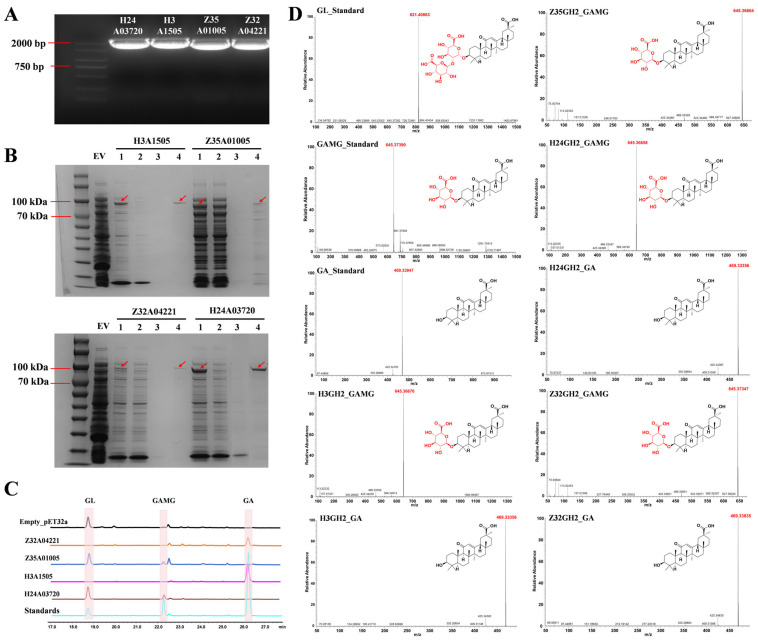
Expression and identification of the catalytic activity of *β*-glucuronidase in a prokaryotic expression system. (**A**) Electrophoresis gel image of PCR amplification for four target *GH2* genes. (**B**) Western blot results of GH2 protein expression and purification (EV: empty vector; 1: crude enzyme; 2: flow-through; 3: wash; 4. eluant). (**C**) The in vitro catalytic activity of four GH2 proteins on glycyrrhizin. (**D**) HPLC-MS/MS identification of products converted from glycyrrhizin by four GH2 proteins in vitro.

The candidate GH2 proteins were expressed in *E. coli*, and in vitro enzymatic assays revealed that H3A1505, Z32A04221 (identical sequence to Z35A05952), Z35A01005, and H24A03720 had catalytic activity for hydrolysis of glycyrrhizin (Figure 3A–C). Among them, H3A1505, Z32A04221, and H24A03720 catalyzed a two-step hydrolysis of GL by sequentially cleaving glucuronide residues to yield GAMG and GA. In contrast, Z35A01005 specifically hydrolyzed one glucuronide unit to produce GAMG only. Based on protein family classification, these active enzymes were designated as H3GH2, Z32GH2 (Z35GH2-1), Z35GH2, and H24GH2. These findings reveal that the four *β*-glucuronidases from the GH2 family of licorice endophytic fungi have diverse glycyrrhizin catalytic activities.

To confirm their catalytic products, HPLC-MS/MS was used to qualitatively analyze the products of in vitro catalysis of glycyrrhizin by four GH2 proteins. Its detection in negative ion mode showed (Figure 3D) that the ionic fragmentation mass fractions of GL, GAMG and GA in the mixed standards after ionization were 821.40863 [M-H]^−^, 645.37300 [M-H]^−^ and 469.33847 [M-H]^−^, respectively. Meanwhile, the 821.39905 [M-H]^−^ ionic feature of glycyrrhizin (substrate) was detected in the CK-GL group; the ionic fragmentation features of the GAMG and GA products were detected simultaneously in the H3GH2, H24GH2 and Z32GH2 catalytic products, whereas only the GAMG (645.36664 [M-H]^−^) was detected in the catalytic product of Z35GH2 (Figure 3C). This provides further evidence that H3GH2, H24GH2, Z32GH2 and Z35GH2 have the catalytic activity to produce the GAMG or GA from glycyrrhizin.

### 2.4. Optimization of β-Glucuronidase Induction and Catalytic Conditions

Optimization of the protein induction conditions revealed distinct expression patterns among the four GH2 enzymes. H24GH2 showed elevated expression at 0.5 and 1.0 mM IPTG, while Z32GH2 and H3GH2 had relatively higher expression at 0.1 and 0.5 mM IPTG. Z35GH2 exhibited increased expression at both 0.1 and 1.0 mM IPTG (Figure S6A). Temperature optimization further showed that H3GH2 and H24GH2 were robustly expressed across all four tested temperatures, Z32GH2 expressed best at 16 °C, 20 °C, and 28 °C (lowest at 24 °C), and Z35GH2 had highest expression at 16 °C (Figure S6B). Therefore, 0.1 mM IPTG was selected for Z32GH2, H3GH2, and Z35GH2 induction, and 0.5 mM for H24GH2, and the optimal induction temperature for all four proteins was determined to be 16 °C.

**Figure 4:**
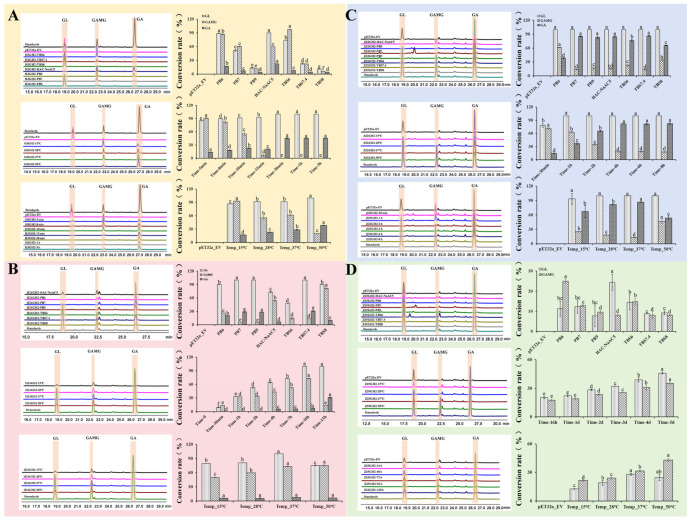
Optimization of catalytic reaction conditions for target GH2 proteins. (**A**) H3GH2. (**B**) H24GH2. (**C**) Z32GH2. (**D**) Z35GH2. Each panel shows glycyrrhizin conversion rates and product (GAMG or GA) yields under varying buffer systems, temperatures, and reaction times. Data are presented as means ± standard error (*n* = 3). The significance of differences in the same measurement parameter for various groups was assessed using one-way ANOVA analysis, with lowercase letters indicating *p* < 0.05.

Protein concentrations, quantified via BCA assay (Y = 0.0015X + 0.0784, R^2^ = 0.9987), were 926.07 μg/mL (H3GH2), 751.51 μg/mL (H24GH2), 984.52 μg/mL (Z32GH2), and 303.73 μg/mL (Z35GH2). Equal protein amounts were then used to evaluate catalytic efficiency under varying buffer pHs, temperatures, and lengths of time. The conversion rate of glycyrrhizin by equal amounts of GH2 protein under different reaction conditions was examined, and it was found that H3GH2 had the highest GL conversion (90.08%) and GA generation (22.27%) in HAC-NaAc5 buffer, and that 100% conversion of GL could be achieved when H3GH2 was reacted for 30 min in this condition (Figure 4). Moreover, the H3GH2 protein had the highest conversion (100%) to GL and GA production (38.45%) at 50 °C under the same conditions (Figure 4A). H24GH2 showed 100% conversion of GL in PB7, PB9 and TBS7.4 (100%) buffers, with the highest GA yield (31.05%) in TBS7.4 buffer. Meanwhile, the 100% conversion of GL could be achieved by H24GH2 in TBS7.4 buffer for 10 h, and the highest GL conversion efficiency (100%) and GA production rate (7.05%) were achieved at 37 °C (Figure 4B). The conversion of GL by Z32GH2 was 100% in all seven different pH buffers, with the highest GAMG production rate (61.43%) in PB6 buffer and the highest GA production rate (86.34%) in TBS7.4 buffer. Simultaneously, Z32GH2 had 100% conversion of GL at 28, 37 and 50 °C, and the highest GA production rate (86.96%) was achieved at 37 °C (Figure 4C). Z35GH2 showed higher GL conversion under acidic (PB6, HAC-NaAC5 and TBS6) and neutral conditions (PB7) than alkaline conditions (PB9, TBS7.4 and TB8), and its GL conversion (24.49%) was highest in HAC-NaAC5 buffer, and the GAMG yield was highest (25.00%) in PB6 buffer. Moreover, Z35GH2 was catalyzed for a longer time than the other three GH2 proteins, with its GL conversion and GAMG production rates of 45.91% and 35.34% in the reaction for 5 d, and its GL conversion (28.09%) and GAMG yield (43.01%) were highest at 37 °C and 50 °C, respectively (Figure 4D). Taken together, the optimal reaction conditions for H3GH2 were HAC-NaAC5, 15 °C for 0.5 h, TBS7.4, 37 °C, 10 h for H24GH2, PB6, 37 °C, 1 h for Z32GH2, and HAC-NaAC5, 50 °C, more than 5 d for Z35GH2. These findings highlight the distinct catalytic behaviors and optimal conditions of the GH2 *β*-glucuronidases, offering insights into their diverse enzymatic profiles in glycyrrhizin biotransformation.

### 2.5. Validation of the In Vivo Catalytic Activity of the Target β-Glucuronidase

**Figure 5:**
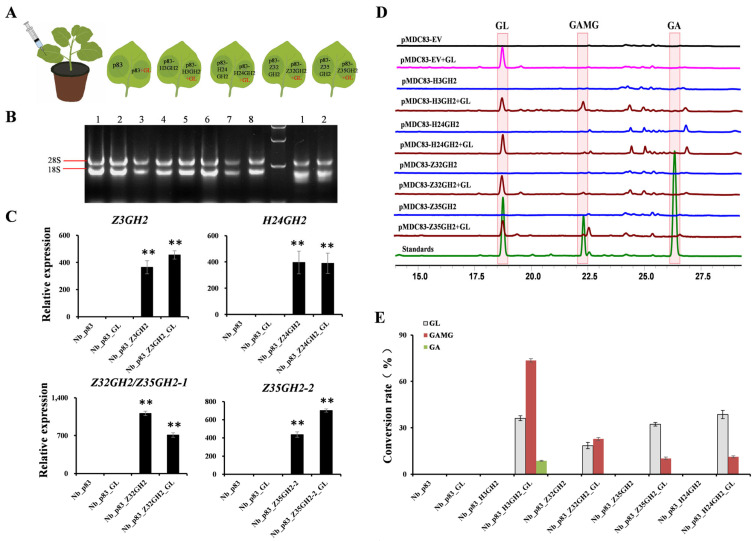
Validation of the in vivo catalytic activity of *β*-glucuronidase. (**A**) Transient expression of the target *GH2* genes in *N. benthamiana* leaves. (**B**) Gel electrophoresis diagram of RNA extracted from *N. benthamiana* leaves. (**C**) Relative expression levels of the *GH2* genes in *N. benthamiana* leaves. (**D**,**E**) Catalytic activity (**D**) and conversion rate (**E**) of four GH2 proteins on glycyrrhizin substrate in *N. benthamiana* leaves. The data presented in the histogram as means ± standard errors (*n* = 3). The asterisk “**” denotes significant difference at *p* < 0.01 among treatments.

To assess in vivo catalytic activity, the recombinant plasmids p83-H3GH2, p83-Z32GH2, p83-Z35GH2, and p83-H24GH2 were introduced into *A. rhizogenes* GV3101 (psoup) for preparing infiltration solution, and then were heterologously expressed in *N. benthamiana* leaves (Figure 5A). The gel electrophoresis result of tobacco leaves total RNA showed complete and specific bands at 28S and 18S positions, indicating that the quality of the extracted RNA was excellent (Figure 5B). qRT-PCR analysis further verified successful heterologous expression of all four GH2 genes in *N. benthamiana* leaves (Figure 5C). HPLC analysis of glycyrrhizin content in transformed leaves showed a significant decrease in GL peak area compared to the control, indicating active glycyrrhizin conversion by the expressed proteins (Figure 5D). In particular, p83-H3GH2-infiltrated leaves exhibited conversion rates of 73.58% for GAMG and 8.75% for GA, confirming its full two-step hydrolytic activity in vivo. In contrast, p83-Z32GH2, p83-Z35GH2, and p83-H24GH2 expression led to accumulation of GAMG only, with GL conversion rates of 18.55%, 32.24%, and 38.64%, respectively. These results demonstrated that H3GH2, Z32GH2, Z35GH2 and H24GH2 proteins from licorice endophytic fungi retain their glycyrrhizin-transforming activity in vivo and can be functionally expressed in heterologous plant systems.

### 2.6. Expression Patterns of β-Glucuronidase Genes Induced by Glycyrrhizin

The expression patterns of *H3GH2*, *H24GH2*, *Z32GH2*, and *Z35GH2* genes in endophytic fungi co-cultured with different concentrations of glycyrrhizin were further analyzed by qRT-PCR. The results showed that the expression levels of *H3GH2*, *Z32GH2*, and *Z35GH2-1* genes were significantly up-regulated after co-culture with glycyrrhizin (Figure S7). Specifically, the relative expression of *Z32GH2* increased with elevated glycyrrhizin concentration, *H3GH2* peaked at 1 g/L glycyrrhizin, whereas *Z35GH2-1* reached its highest level at 3 g/L. In addition, the *H24GH2* and *Z35GH2* genes showed a decreasing and then increasing expression pattern under different concentrations of glycyrrhizin-induced conditions. It is evident that even the same functional gene, such as *Z32GH2* (*Z35GH2-1*), also showed different glycyrrhizin concentration response patterns in different endophytic fungi of Z32 and Z35. These results suggested that the four *β*-glucuronidase genes with glycyrrhizin hydrolyzing activity were able to show diverse expression response patterns based on the different concentrations of glycyrrhizin.

### 2.7. Analysis of Structure-Activity Differences in GH2 Proteins

The structure, domains and conserved motifs of proteins have an important impact on their function. The four protein models predicted by AlphaFold 3 were individually aligned using Pymol software, and it was found that the RMSD values of H3GH2, Z32GH2, and H24GH2 protein comparisons with the same catalytic type (GL to GAMG and GA) ranged from 0.126 to 0.261 Å, which indicated that they had highly similar structures. And the RMSD values of Z35GH2 with another catalytic mode (GL to GAMG) were found to be 26.365 Å, 30.173 Å, and 29.520 Å compared with H3GH2, Z32GH2, and H24GH2, which indicated that there were distinctive differences in their structures (Figure S8A,B). Annotation analysis of protein conserved domains showed that H3GH2, Z32GH2 and H24GH2 have consistent conserved domains (Glyco_hudro_2_N, Immunoglobulins and Gly_hydro_2_C) and four Glycosyl hydrolase family 2 signatures; however, Z35GH2 has a completely different mannosidase Ig/CBM-like domain and beta-Galactosidase/glucuronidase domain (Figure S8C, Tables S5 and S6). In addition, they also differed in GO annotation, where H3GH2, Z32GH2 and H24GH2 are conserved in biological process (glucuronoside catabolic process (GO:0019391)) and Molecular Function (carbohydrate binding (GO:0030246) and beta-glucuronidase activity (GO:0004566)) were consistent, while Z35GH2 was annotated to glycoprotein catabolic process (GO:0006516) and beta-mannosidase activity (GO:0004567), respectively (Table S5). Further analysis revealed that within 10 search thresholds, H3GH2, Z32GH2 and H24GH2 had highly similar conserved motifs to Z15GH2 and AtGUS, which have been reported to have the same catalytic type, whereas no similar conserved motifs existed for Z35GH2 (Figure S8D). Z35GH2 was found to have one similar conserved motif 8 in further comparison with Cg-GUS and PGUS with the same catalytic type (Figure S8E). These results further illustrated that the relatively conserved protein structures, functional domains, and conserved motifs that proteins of the same catalytic type have in different species determine their similar catalytic activities, and also that variations in these conserved features are key to the differences in catalytic activities of different proteins.

### 2.8. The Effect of Inoculation with Endophytic Fungi on the Accumulation of Active Components in Licorice

**Figure 6:**
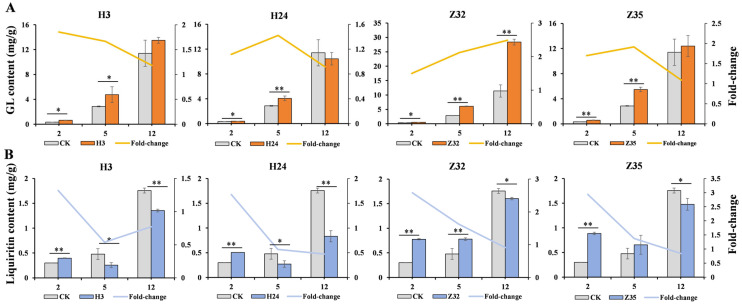
Content of glycyrrhizin (**A**) and liquiritin (**B**) in licorice root inoculated with four endophytic fungi (H3, H24, Z32 and Z35). The primary vertical axis (left) represents the content of the target active compound in licorice root, and the secondary vertical axis (right) represents the fold change in the content of the target compound in the treatment group compared with the control group (CK). The “*” and “**” represent *p* < 0.05 and *p* < 0.01.

Quantitative analysis of key bioactive constituents in licorice root revealed that inoculation with four endophytic fungi significantly elevated glycyrrhizin content across all root samples, with varying degrees of enhancement observed among fungal strains (Figure 6A). Conversely, liquiritin content showed significant elevation only in the roots of 2-month-old licorice roots (Figure 6B). Specifically, compared with the control group, the glycyrrhizin content in the inoculated licorice ranged from 1.09 to 2.48 times, and the liquiritin content in 2-month-old inoculated licorice ranged from 1.32 to 2.94. Comparative analysis of glycyrrhizin content among different treatment groups showed that in 2-month-old licorice roots, the H3 inoculation group exhibited the highest increase (85.37%), while the H24 inoculation group showed the lowest increase (11.61%). In 5-month-old and 12-month-old licorice roots, the Z32 strain exhibited the strongest promotion effect on glycyrrhizin accumulation, with significant increases of 112.42% and 148.91% compared with the control group, respectively. Except for 5-month-old licorice inoculated with Z32 and Z35 strains, the liquiritin content in 5-month-old and 12-month-old licorice roots showed a decreasing trend after endophytic fungal inoculation. These results demonstrate that the four endophytic fungi (H3, H24, Z32, and Z35) not only possess the capacity for glycyrrhizin biotransformation but also significantly promote glycyrrhizin accumulation in licorice roots following inoculation, with the promotion effect being closely related to the fungal strain and the growth stage of the host licorice.

## 3. Discussion

Glycoside hydrolases are pivotal enzymes that cleave glycosidic bonds in glycosides, playing essential roles in carbohydrate metabolism across various organisms [22]. Among them, *β*-glucosidases are capable of hydrolyzing terminal glycosidic linkages in glycosides and oligosaccharides, contributing to diverse metabolic processes in both microbes and plants [23]. For instance, Mamma [24] identified both *β*-glucosidases G(I) and G(II) from *Penicillium*, which can catalyze the hydrolysis of flavonoid compounds with seven-position glycosylation, resulting in efficient deglycosylation. In addition, *β*-glucosidases from microorganisms also play a key catalytic role in the biotransformation or hydrolysis of various plant secondary metabolites (PSMs), including ginsenosides [25], icariin [26], soybean glycosides [27], and glycyrrhizin [21]. In particular, glycyrrhizin, a triterpenoid saponin abundant in licorice root, features two glucuronic acid moieties linked to the C-3 position of glycyrrhizin via *β*-1,2-glycosidic bonds. Its hydrolysis products, GAMG and GA, exhibit significantly enhanced pharmacological activity and bioavailability [6,8]. Previous studies have reported that members of the GH2 and GH79 families are capable of catalyzing glycyrrhizin hydrolysis to generate GAMG and GA [17,18,20,28]. Endophytic fungi, which reside asymptomatically within plant tissues, may utilize such enzymatic activities to metabolize host PSMs, serving as a key chemical adaptation strategy to colonize and persist in the host environment [21].

In this study, we initially screened 16 licorice endophytic fungi for their ability to metabolize glycyrrhizin, and further identified nine strains exhibiting significant *β*-glucosidase activities during co-culture with glycyrrhizin by GUS staining. A total of 93 GH2-family glycoside hydrolase genes were identified from the genomes of these nine strains. Based on systematic bioinformatic analysis, domain structure prediction, and ligand-binding site analysis, 12 candidate GH2 proteins were selected for further functional validation. Four of these candidate proteins (H3GH2, H24GH2, Z32GH2, Z35GH2) were successfully validated through prokaryotic expression in *E. coli* and in vitro enzyme activity assays. Notably, H3GH2, H24GH2, and Z32GH2 exhibited tetrameric structures resembling the *β*-glucuronidase from *A. oryzae* Li-3 [16], underscoring the critical role of quaternary structure in maintaining the catalytic function. In terms of biotransformation types, H3GH2, H24GH2 and Z32GH2 displayed similar catalytic patterns to the GUS identified from the rhizosphere fungus *A. terreus* Li-20 [18] and the licorice endophytic fungus Z15 [21], as all three enzymes were capable of hydrolyzing GL to produce both GAMG and GA via a two-step pathway. In contrast, Z35GH2 exhibited a single-step catalytic mode similar to TpGUS79A in the rice endophytic fungus *C. globosum* DX-THS3 [28], hydrolyzing GL only to GAMG. However, a key distinction is that TpGUS79A belongs to the GH79 family, while Z35GH2 is a member of the GH2 family, highlighting the functional diversity of glycoside hydrolases across different GH families in mediating glycyrrhizin hydrolysis. Furthermore, the present study confirmed that H3GH2, H24GH2, Z32GH2 and Z35GH2 proteins have relatively stable catalytic activities to GL in buffers, with pH ranging from 5 to 7.4 and temperature between 15 °C and 50 °C. This stability is consistent with the *β*-GUS enzymes from *E. coli* [17] and filamentous fungi [29], suggesting that these four GH2 proteins are well-suited for industrial-scale biotransformation of glycyrrhizin. Their differential substrate selectivity and product profiles also highlight the structural diversity and functional adaptability of fungal GH2 enzymes, providing valuable mechanistic insights into how endophytic fungi metabolize host-derived specialized metabolites for ecological adaptation.

*Agrobacterium*-mediated transformation has emerged as a simple and effective method for delivering target genes into plant cells to express recombinant proteins and perform functional validation [30,31]. *Agrobacterium*-mediated transient transformation in *Nicotiana benthamiana* also has been widely used to study the function of heterologous genes. Previous studies illustrated the GV3101 strain yields the highest recombinant protein levels in *N. benthamiana* leaves compared to LBA4404 and C58C1 and wild-type strains at6, at10, at77, and A4 [32]. Zhang et al. [33] successfully validated the catalytic activity of littorine synthase by constructing a GV3101-mediated transient transformation system in *N. benthamiana* and exogenously adding tropine and hydroxyphenyllactic acid. Consistent with these reports, in the present study, the four GH2 genes (*H3GH2*, *H24GH2*, *Z32GH2* and *Z35GH2*) identified from licorice endophytic fungi were recombinantly inserted into the plant expression vector pMDC83 and heterologously expressed in *N. benthamiana* leaves through the *A. rhizome* GV3101-mediated transformation method. Upon exogenous glycyrrhizin infiltration, these recombinant proteins exhibited clear in vivo hydrolytic activity, as evidenced by the significant reduction in glycyrrhizin levels and the detection of GAMG and GA products. Notably, H3GH2 exhibited the highest conversion rates to GAMG and GA, which is consistent with its strong catalytic performance in in vitro assays. The significant reduction in glycyrrhizin substrate content and the successful detection of its hydrolysis products in *N. benthamiana* leaves further demonstrate that plant tissues can serve as efficient platforms for evaluating the in vivo enzymatic activity of fungal glycoside hydrolases. In addition, qRT-PCR analysis revealed that the expression of the target GH2 genes (*H3GH2*, *H24GH2*, *Z32GH2* and *Z35GH2*) was induced by glycyrrhizin in a concentration-dependent manner. These findings deepen our understanding of the biochemical pathways through which endophytic fungi biotransform plant saponins, while also providing practical enzymatic tools for the targeted and eco-friendly production of high-value glycyrrhizin derivatives.

Importantly, the differential catalytic activities of these four GH2 enzymes are closely correlated with the distinct effects of their corresponding host endophytic fungi on glycyrrhizin accumulation in inoculated licorice (Table S7). Specifically, Z32GH2 showed efficient and stable catalytic activity both in vitro and in vivo, and the Z32 strain conferred the most pronounced enhancement of glycyrrhizin accumulation in licorice roots at both 5 and 12 months post infection. In contrast, H24GH2 exhibited relatively weak catalytic efficiency to glycyrrhizin, and inoculation with H24 strain resulted in the weakest promoting effect on host glycyrrhizin accumulation among the four tested strains. Correlation analysis further validated that the transcript levels of glycyrrhizin-hydrolyzing GH2 genes in strains H3, Z32, and Z35 were significantly positively correlated with glycyrrhizin content in inoculated licorice (*p* < 0.05). Conversely, a weak and statistically non-significant positive correlation was detected between *H24GH2* gene expression and host glycyrrhizin accumulation (*r* = 0.122, *p* = 0.754). Collectively, these findings preliminarily suggest a potential synergistic relationship between the glycyrrhizin catalytic activity of endophytic fungal GH2 enzymes and their regulatory function in modulating host glycyrrhizin biosynthesis in vivo. Although all strains used in this study were endophytes originally isolated from healthy licorice root, and no obvious symptoms were observed during inoculation of the licorice, further investigation is required to determine whether these strains are pathogenic to the host and to assess their colonization efficiency. In summary, this study provides a novel insight into the role of endophytic fungi in regulating the development of medicinal plant quality formation, and lays the theoretical foundation for the targeted application of endophytic fungi in the genetic improvement and quality enhancement of licorice.

## 4. Materials and Methods

### 4.1. Identification of Glycyrrhizin Conversion Activity

Endophytic fungi isolated from licorice roots were maintained in the laboratory of the School of Chinese Materia Medica, Beijing University of Chinese Medicine (Beijing, China). Thirty-three strains were inoculated onto minimal medium supplemented with 3 g/L glycyrrhizin (G0150, Tokyo Chemical Industry, Shanghai, China) as the sole carbon source using the mycelial inoculation method, while an equal amount of glucose carbon source served as the control, and they were incubated at 28 °C in darkness for 7 days. Strains showing comparable growth to the glucose control were selected as candidate glycyrrhizin-metabolizing fungi. Spores were collected from 7-day-old cultures using 5 mL of sterile water and filtered to prepare suspensions at approximately 1 × 10^8^ CFU/mL. Aliquots (500 µL) of spore solution were incubated into 50 mL of PDB liquid medium and cultured at 28 °C, 180 rpm for 2 days. Glycyrrhizin (final concentration 1 mg/mL) was then added, and fermentation continued for 5 days. Control groups included uninoculated PDB and PDB with glycyrrhizin only. All culture solutions were extracted with ethyl acetate, concentrated to dryness, fully reconstituted in methanol, and analyzed using the HPLC method [21] to further characterize the metabolic activity of the endophytic fungi on glycyrrhizin.

### 4.2. Species Identification and β-Glucuronidase Induced-Expression of Endophytic Fungi

For taxonomic identification, target strains were cultured on PDA for 7 days at 28 °C. Morphological characteristics were recorded. Genomic DNA was extracted and ITS sequences amplified using primers universal ITS1and ITS4. Sequences were aligned using NCBI BLAST (https://pubmed.ncbi.nlm.nih.gov/), and phylogenetic trees were constructed via the neighbor-joining method with 1000 bootstrap replicates using MEGA (v.11.0).

To investigate GUS activity in response to glycyrrhizin, fungal plugs (5 mm × 5 mm) were inoculated into 1/5 PDB medium containing 0, 1, 3, or 5 g/L glycyrrhizin in 12-well plates, following the co-culture method [34]. Three biological replicates were performed for each treatment. After 5 days of incubation, mycelia were stained overnight at 37 °C with GUS staining solution. GUS expression was assessed based on the intensity of blue coloration.

### 4.3. Draft Genome Sequencing and GH2 Candidate Genes Screening

The total genomic DNA of nine selected strains (Z9, Z11, Z22, Z25, Z32, Z35, H3, H16, and H24) was quality-tested according to the GUS staining results, and DNA sequencing libraries were constructed for each of the nine strains in compliance with the requirements of the NEB Next^®^ Ultra™ DNA Library Preparation Kit (New England Biolabs, Ipswich, MA, USA). The quality of the constructed DNA libraries was assessed by preliminary and accurate quantitative assessment using Qubit 2.0 and Q-PCR methods. Raw data and filtered validated data (Clean data) were acquired by sequencing the whole genomes of nine target endophytic fungi on the Illumina NovaSeq34 PE150 platform and further obtained the coding genes annotation information by assembly and predictive analyses of the genomes. Annotation analysis of the encoded genes was performed using the Carbohydrate-Active CAZymes Database [35] to further obtain the relevant catalytic enzymes involved in carbohydrate biosynthesis, degradation and modification.

Glycoside hydrolase family 2 (GH2) genes were screened from the predicted genomes of these strains. The phylogenetic tree of GH2 proteins was constructed using MEGA 11.0 software, which integrates the reported non-licorice endophytic fungal sources (JF894133, MF282011, MN207130, JQ897940, and JH10433) and licorice endophytic fungal sources (Z6GH2 and Z15GH2) of GH2 proteins with glycyrrhizin-catalyzed activities as reference. The bioinformatics of the candidate GH2 proteins were further analyzed by online platforms, where MEME (https://meme-suite.org/meme/) (accessed on 1 July 2024) was used to predict their conserved functional motifs to identify their potential catalytic activities, ExPaSy (https://www.expasy.org/) was used to analyze its physicochemical properties, HMMER (https://www.ebi.ac.uk/Tools/hmmer/search/hmmscan) (accessed on 1 July 2024) was used to identify its family of characteristically conserved structural domains, and InterPro (https://www.ebi.ac.uk/interpro/) (accessed on 1 July 2024) was used to predict and annotate its functional domains and GO classification. The online websites of AlphaFold 3 [36] (https://golgi.sandbox.google.com/about) (accessed on 1 August 2024) and the SWISS-MODEL (https://swissmodel.expasy.org/) (accessed on 1 August 2024) were used to predict and analyze the 3D structural models of the candidate GH2 proteins. The activity pockets of the target GH2 proteins were predicted by the Proteins Plus Server (https://proteins.plus) (accessed on 1 August 2024) platform, while the optimal docking models of the GH2 protein and glycyrrhizin ligand were constructed separately using AutoDock 4.2 software. The interaction patterns of key amino acid residues between the GH2 protein and the glycyrrhizin ligand and the characteristics of the distribution of intermolecular forces were determined based on Pymol’s visualization analysis to elucidate the potential activity of candidate GH2 proteins to catalyze glycyrrhizin at the atomic level.

### 4.4. β-Glucuronidase Gene Cloning and In Vitro Catalytic Activity of Glycyrrhizin

To validate the catalytic functions of GH2 candidates identified in silico, selected genes were cloned and expressed in a prokaryotic system. Total RNA was extracted from nine target strains using RNAiso Plus reagent (Takara, Janpan), and their high-quality cDNAs were synthesized by reverse transcription according to PrimeScript™ II 1st Strand cDNA Synthesis Kit (Vazyme, China). Specific primers (15–20 bp homology arm sequence added at the 5′ end) were designed (Table S1) and used for amplification via 2 × Phanta Flash Master Mix high-fidelity DNA polymerase (Vazyme, China). The purified PCR products were recombined into the linear pET-32a(+) vector using the ClonExpress II One Step Cloning kit and transformed into Trans-T1 cells for positive colony screening via PCR with specific primers (F: 5′-TAATACGACTCACTATAGGG-3′, R: 5′-GCTAGTTATTGCTCAGCGG-3′). Verified constructs were sequenced and aligned using DNAMAN software (version 6), and confirmed plasmids were subsequently transformed into BL21(DE3) for protein expression.

For expression induction, positive transformants were grown in LB liquid medium containing 100 mg/L ampicillin (1% *v*/*v* inoculation) and incubated at 37 °C with shaking 160 rpm until the OD_600_ reached approximately 0.6. Protein expression was induced by the addition of 0.2 mmol/L IPTG, with cells harboring the empty pET-32a(+) vector serving as negative controls. Following induction, cells were harvested by centrifugation, and recombinant proteins were purified according to the BeyoGold™ His-tag Purification Resin kit (Beyotime, China) and detected by SDS-PAGE electrophoresis. To assess enzymatic activity, 200 µL of glycyrrhizin substrate and 20 µL of GH2 protein solution was formulated and the catalytic activity of the candidate proteins towards glycyrrhizin was identified by HPLC method [21] after reaction for 12 h at 37 °C, while the pET-32a(+) empty vector induced solution was used as a negative control. Additionally, reaction products were qualitatively identified using high-performance liquid chromatography–tandem mass spectrometry (HPLC-MS/MS) assay [21].

### 4.5. Optimization of In Vitro Catalytic Reaction Conditions

To optimize the expression and catalytic reaction conditions, bacterial cultures were subjected to a range of IPTG concentrations (0.1, 0.2, 0.5, 1.0 mmol/L) and induction temperatures (16 °C, 20 °C, 24 °C, 28 °C). The concentration of GH2 recombinant protein solution under optimal induction conditions was further determined by BCA protein quantification. Glycyrrhizin conversion rates and product yields were evaluated under different reaction conditions, including buffer systems (HAc-NaAc, pH 5.0; Tris-HCl, pH 6.0, 7.4, and 8.0; phosphate buffer, pH 6.0, 7.0, and 9.0), incubation temperatures (15 °C, 28 °C, 37 °C, 50 °C) and reaction times (0.5, 1, 2, 4, 8, 12 h). Reactions were examined by HPLC to determine the optimal reaction conditions for the catalytic activity of each GH2 protein.

### 4.6. Catalytic Activity Validation of β-Glucuronidase in Nicotiana benthamiana

The *GH2* target genes (*H3GH2*, *H24GH2*, *Z32GH2* and *Z35GH2*) were cloned and recombined into the plant expression vector pMDC83 (p83). Specific primers (p83_F: 5′-GTTAATTAAGAATTAGCTTGCATG-3′ and p83_R: 5′-GAGCGGATAACAATTTCACACAGG-3′) were used to perform PCR identification of the cloned strains, while positive clones were screened for sequencing analysis. The positive recombinant plasmids (p83-H3GH2, p83-H24GH2, p83-Z32GH2 and p83-Z35GH2) were extracted and further transformed into *Agrobacterium tumefaciens* GV3101 to prepare the infiltration solution, respectively. Transient expression in *N. benthamiana* leaves was carried out via *Agrobacterium*-mediated infiltration, following the protocol of Zhang [33]. After 48 h of infiltration, leaves were injected with 2 mM glycyrrhizin to assess in vivo bioconversion. Quantitative RT-PCR (qRT-PCR) was used to detect the heterologous expression levels of the target genes in *N. benthamiana* leaves, while HPLC technique was used to verify the in vivo catalytic glycyrrhizin activity of the target genes. Five biological replicates were performed for each treatment using the empty vector (pMDC83) and wild-type *N. benthamiana* as negative controls, respectively.

### 4.7. qRT-PCR Analysis

To investigate the transcriptional response of GH2 genes to glycyrrhizin concentration, strains H3, H24, Z32 and Z35 were inoculated onto PDA medium containing 0, 1, 3 and 5 mg/mL glycyrrhizin and cultured for 7 d at 28 °C under dark conditions. The total RNA of the cultured strains was extracted by the Trizol method, and RNA quality was identified by 1% agarose gel electrophoresis and ultra-low-volume nucleic acid analyzer. The 2 µg of high-quality RNA was further extracted for reverse transcription to synthesize cDNA. Specific primers (Table S1) were designed based on the target gene sequences, and the reaction systems were prepared separately using the cDNA of the target strains as templates for qRT-PCR analysis. The *H3Actin*, *H24SSD*, *Z32SSD* and *Z35SSD* genes were used as reference genes. And the relative expression of the target GH2 genes was calculated using the 2^−ΔΔCT^ method [37] in order to analyze the expression patterns under different glycyrrhizin concentrations.

### 4.8. Inoculation of Licorice with Endophytic Fungi and Content of Active Components

Four fungal strains (H3, H24, Z32 and Z35) were re-inoculated into licorice to investigate the interaction between endophytic fungi possessing stable glycyrrhizin bioconversion activity and licorice, and to elucidate the effect of endophytic fungal re-infection on the accumulation of active components in licorice. Spore suspensions (2 × 10^6^ CFU/mL) of strains H3, H24, Z32 and Z35 were prepared and inoculated onto surface-sterilized licorice seeds, with sterile water treatment serving as the control group. The treated seeds were cultivated in an artificial climate chamber until germination, then transplanted into nutrient substrate and grown under identical environmental conditions, receiving weekly watering throughout the period. Licorice roots (the primary site of glycyrrhizin accumulation) of inoculated licorice at different growth stages (2, 5 and 12 months) were sampled after, and analyzed for liquiritin and glycyrrhizin content using HPLC with the external standard method [21]. Each treatment group comprised three biological replicates. In addition, Pearson’s method was used to investigate the potential regulatory relationship between the expression levels of the target GH2 gene and the glycyrrhizin content in licorice roots inoculated with endophytic fungi.

## 5. Conclusions

This study screened nine licorice endophytic fungi with both glycyrrhizin biotransformation activity and glycyrrhizin-inducible *β*-glucuronidase activity. Four GH2 family *β*-glucuronidases (H3GH2, H24GH2, Z32GH2, and Z35GH2) were identified and their catalytic activity in hydrolyzing glycyrrhizin via two distinct pathways was validated through *E. coli* prokaryotic expression and *Rhizobium*-mediated transient expression in *N. benthamiana.* qRT-PCR analysis demonstrated that expression of these GH2 genes is induced by glycyrrhizin in a concentration-dependent manner, providing a molecular basis for the regulatory mechanism of glycyrrhizin biotransformation in endophytic fungi. Collectively, this study systematically illuminates the molecular mechanism underlying endophytic fungal *β*-glucuronidase-catalyzed glycyrrhizin biotransformation, and supplies novel and efficient enzymatic tools for the targeted preparation of high-value bioactive glycyrrhizin derivatives. These findings also lay the theoretical and technical foundation for the precise regulation of licorice quality formation through beneficial endophytic fungi, and expand the current understanding of endophyte–host metabolic crosstalk in medicinal plants’ specialized metabolism.

## Data Availability

The original contributions presented in this study are included in the article/Supplementary Materials. Draft genomic sequence data for nine endophytic fungi are available via the NCBI database (PRJNA1470062) and will be publically released on 30 July 2026.

## Supplementary Materials

The following supporting information can be downloaded at https://www.mdpi.com/article/10.3390/ijms27125444/s1.
