# Supplementary material for: Differential Biotransformation of Glycyrrhizin by Licorice-Derived Endophytic Fungi and Accumulation-Promoting Effects of Fungal Inoculation
Source: Int J Mol Sci. 2026 Jun 16;27(12):5444. doi: 10.3390/ijms27125444 (PMC13299809; doi:10.3390/ijms27125444)
Supplement: Supplementary file 1 [file ijms-27-05444-s001.zip › ijms-4265754-supplementary.pdf]

# Differential Biotransformation of Glycyrrhizin by Licorice-derived Endophytic Fungi and Accumulation-Promoting Effects of fungal Reinfection

**Xin Zuo[a,b], Guangxi Ren[a], Dan Jiang[a, *], Chunsheng Liu[a, *]**

[a] Beijing University of Chinese Medicine, Beijing, China, 102488

[b] Luoyang Central Hospital Affiliated to Zhengzhou University, Luoyang, China, 471000

[*] **Corresponding author:** max_liucs@263.net (C.S. Liu), jiangdan1027@163.com (D. Jiang);

## Supplementary materials Legends:

**Figure S1. Identification of metabolic activity on glycyrrhizin by licorice endophytic fungi.**

**Figure S2. Identification of glycyrrhizin conversion function and *β*-glucuronidase activity of licorice endophytic fungi.**

**Figure S3. NR functional annotation of endogenous fungal-encoded genes**

**Figure S4. CAZy annotation results of nine candidate licorice endophytic fungi based on genome sequencing.**

**Figure S5. Comparative analysis for conserved motifs of GH2 proteins from nine licorice endophytic fungi.**

**Figure S6. Western blot identification of four target GH2 proteins under different induction temperatures (A) or IPTG concentrations (B).**

**Figure S7. Expression patterns of the target *GH2* genes at different glycyrrhizin concentrations.**

**Figure S8. Predictive analysis of structural activity differences in GH2 proteins.**

**Table S1. Primer sequences used for PCR amplification.**

**Table S2. Whole-genome assembly of nine licorice endophytic fungi.**

**Table S3. 3D structure prediction results for 12 GH2 candidate proteins.**

**Table S4. Molecular docking scores of GH2 candidate proteins with glycyrrhizin ligands.**

**Table S5. Conserved structural features and functional predictionsof GH2 proteins.**

**Table S6. Glycosyl hydrolase family 2 signatures.**

**Table S7. Correlation between endogenous fungal glycyrrhizin hydrolyzing *GH2* gene expression levels and glycyrrhizin content in reinfected licorice roots.**

**Figure S1. Identification of metabolic activity on glycyrrhizin by licorice endophytic fungi. (A)** Growth morphology of licorice endophytic fungi on minimal medium with glycyrrhizin. **(B)** Fermentation of licorice endophytic fungi in liquid medium supplemented with exogenous glycyrrhizin. **(C)** The conversion rate of glycyrrhizin by licorice endophytic fungi during liquid fermentation.

A

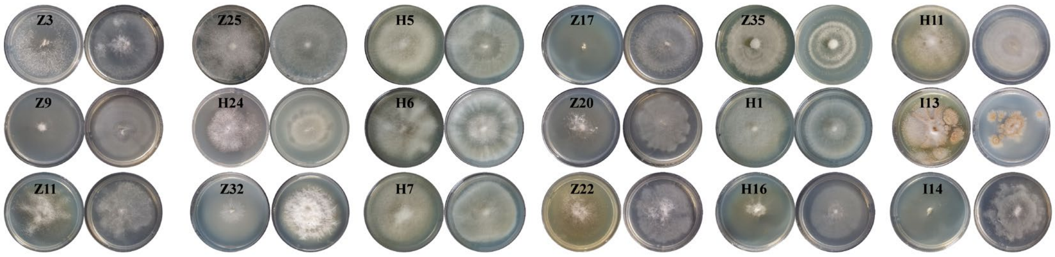


B

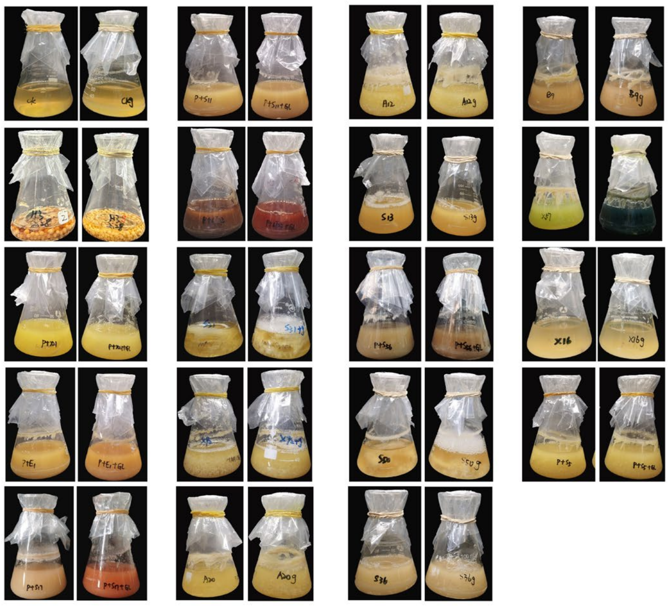

C

Conversion of GL to strains in liquid fermentation

| Strain | Conversion rate （%） |
|---|---|
| CK | 0 |
| H3 | 92.72 |
| Z9 | 57.37 |
| Z11 | 78.87 |
| Z17 | 0 |
| Z20 | 0 |
| Z22 | 100 |
| Z25 | 100 |
| H24 | 100 |
| Z32 | 100 |
| Z35 | 100 |
| H1 | 100 |
| H16 | 100 |
| H5 | 100 |
| H6 | 100 |
| H7 | 29.46 |
| H11 | 97.56 |
| I13 | 28.45 |
| I14 | 67.61 |

**Figure S2. Identification of glycyrrhizin conversion function and *β*-glucuronidase activity of licorice endophytic fungi.** **(A)** Growth morphology of licorice endophytic fungi on minimal medium with glycyrrhizin. b-c: Growth morphology **(B)** and *β*-glucuronidase activity **(C)** of licorice endophytic fungi after co-cultivation with glycyrrhizin.

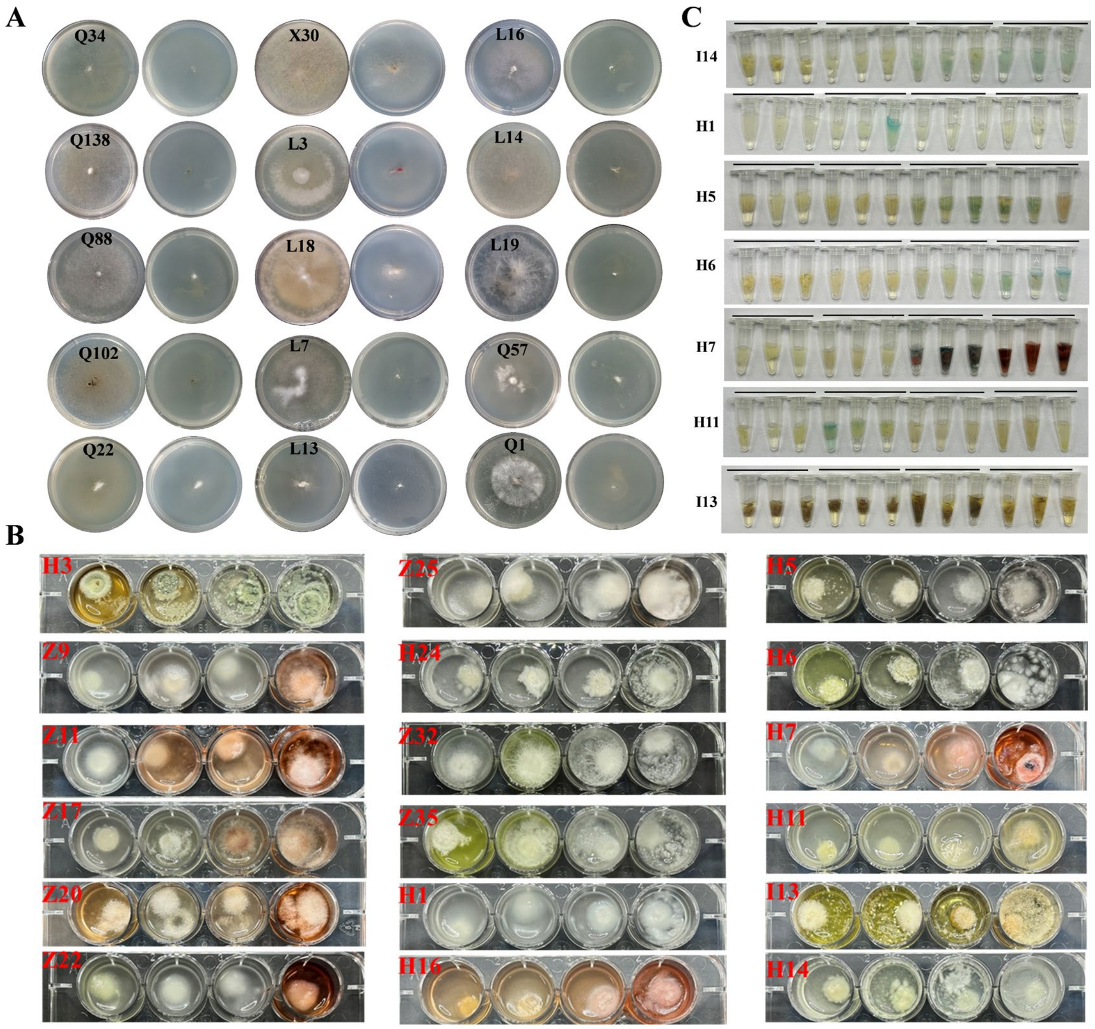

# Figure S3. NR functional annotation of endogenous fungal-encoded genes

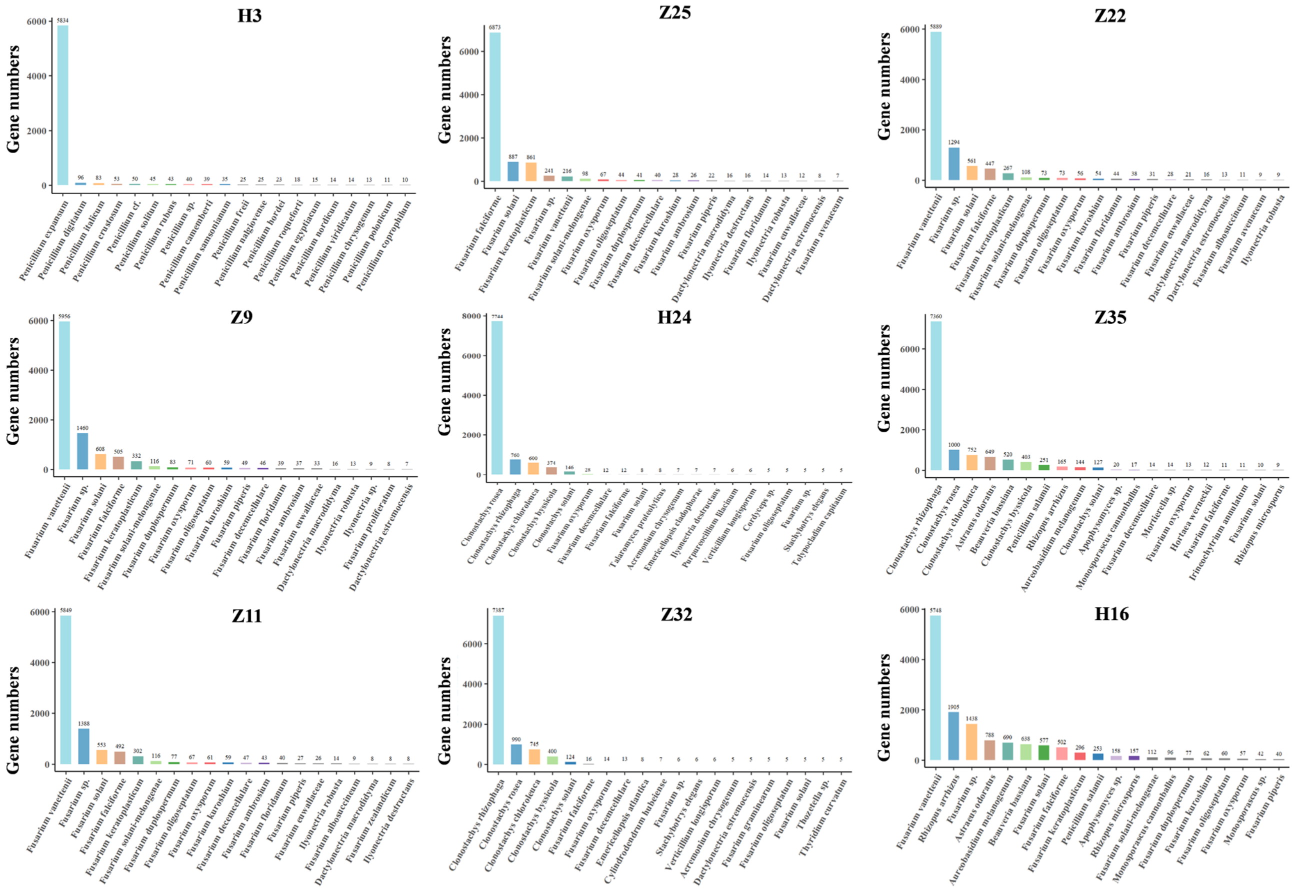

**Figure S4. CAZy annotation results of nine candidate licorice endophytic fungi based on genome sequencing.**

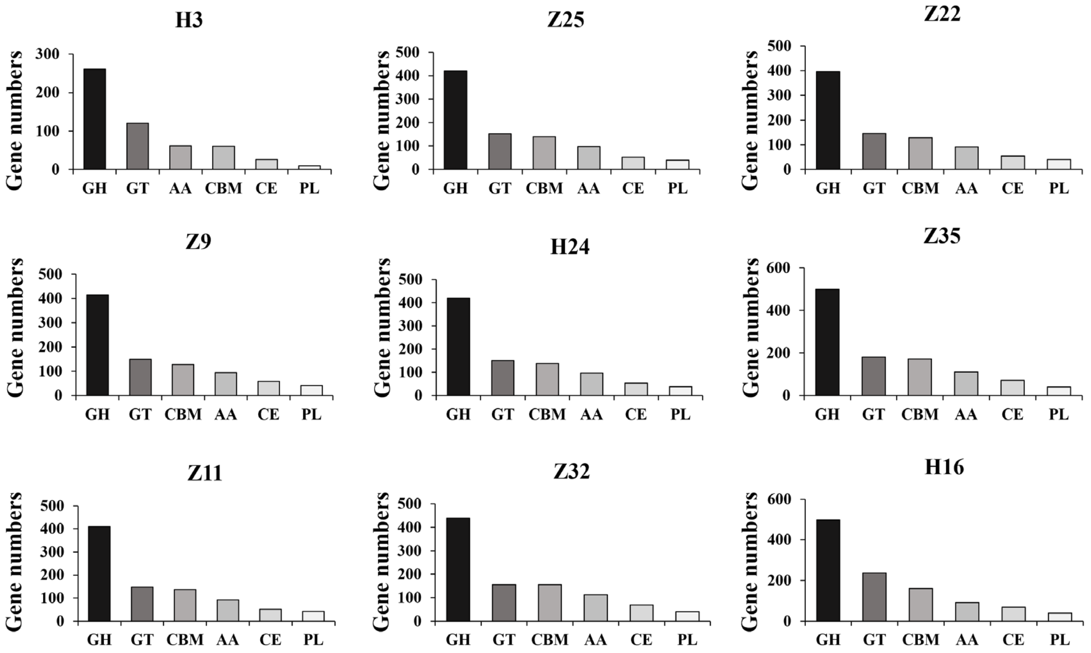

**Figure S5. Comparative analysis for conserved motifs of GH2 proteins from nine licorice endophytic fungi.** **(A)** Z35; **(B)** Z9; **(C)** H3; **(D)** H24; **(E)** Z22; **(F)** Z25; **(G)** Z32; **(H)** H16; **(I)** Z11.

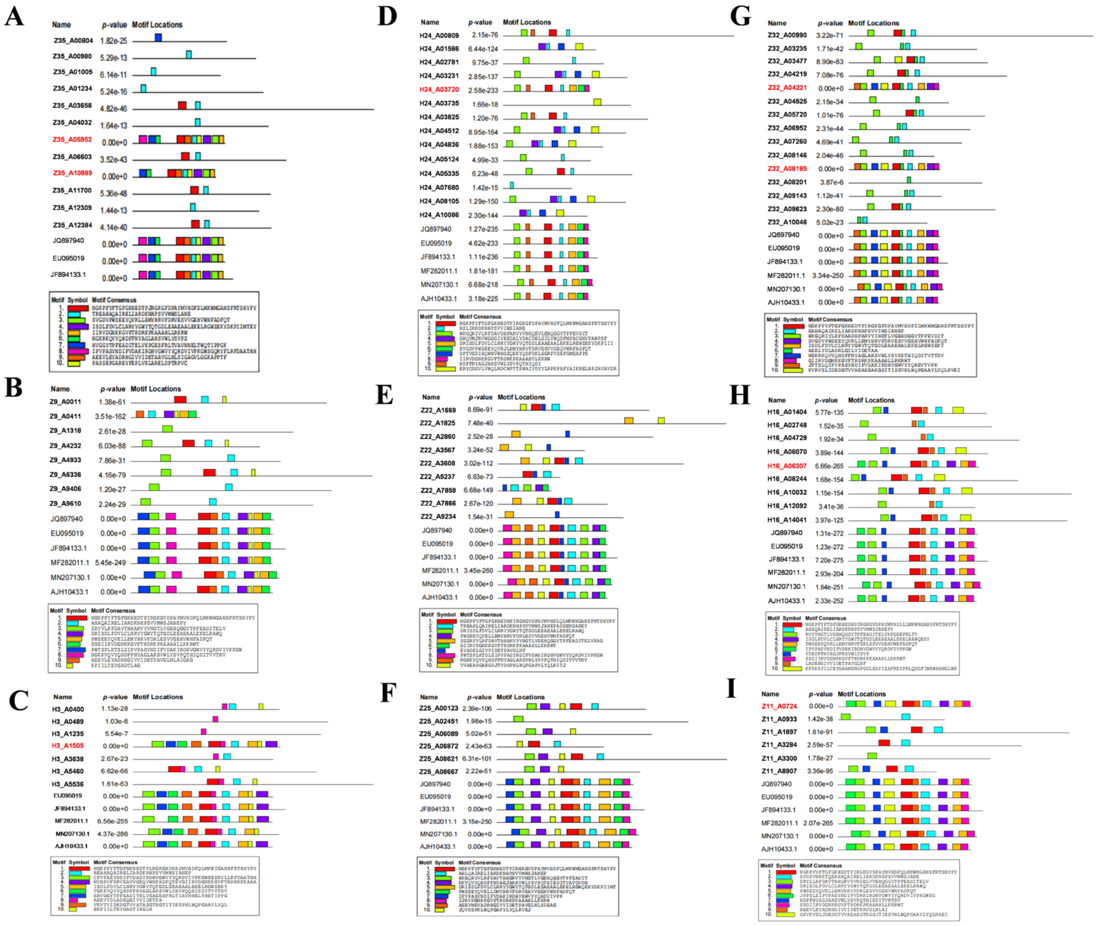

**Figure S6. Western blot identification of four target GH2 proteins under different induction temperatures (A) or IPTG concentrations (B).** EV: empty vector.

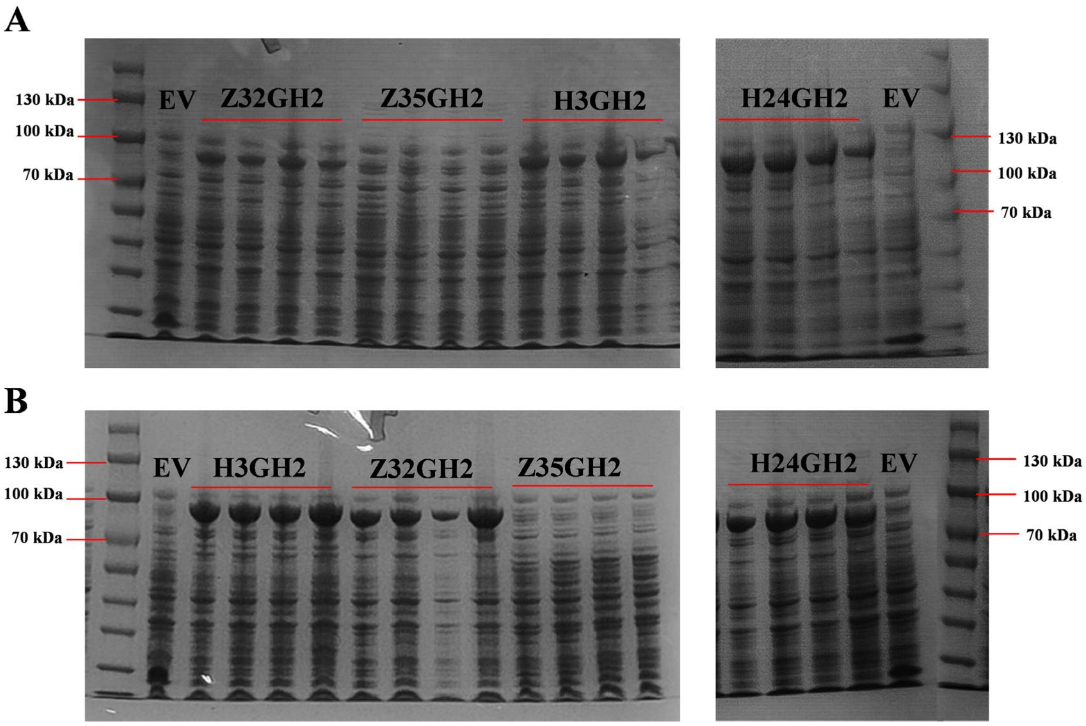

**Figure S7. Expression patterns of the target *GH2* genes at different glycyrrhizin concentrations.** One-way ANOVA was used to assess the level of expression differences between groups, with lowercase letters indicating $p < 0.05$.

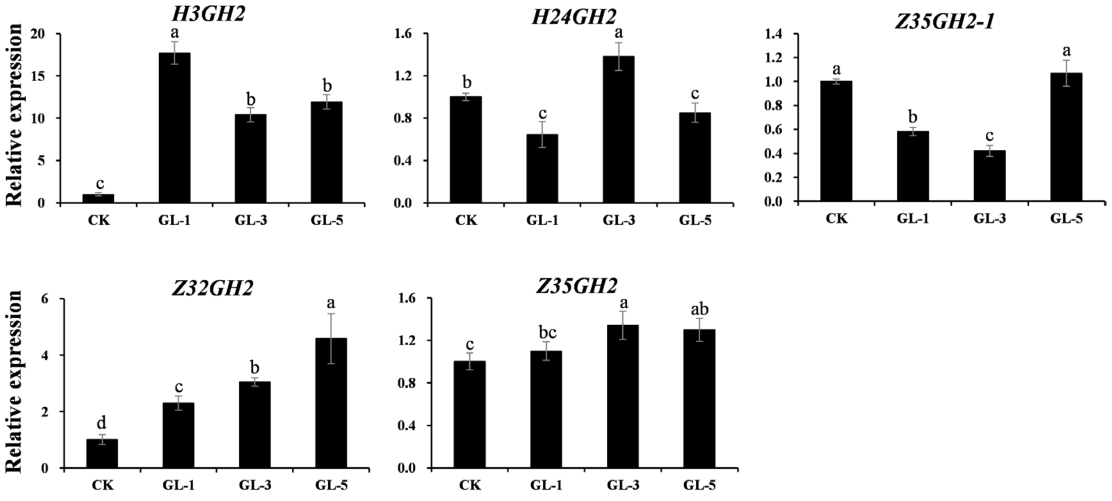

**Figure S8. Predictive analysis of structural activity differences in GH2 proteins. (A)** Comparison of the protein structures of H3GH2, H24GH2, Z32GH2, and Z35GH2. **(B)** RMSD values between different protein structures. **(C)** Conserved domain analysis of H3GH2, H24GH2, Z32GH2, and Z35GH2 proteins. **(D-E)** Similar conserved motif analysis of H3GH2, H24GH2, Z32GH2, and Z35GH2 proteins.

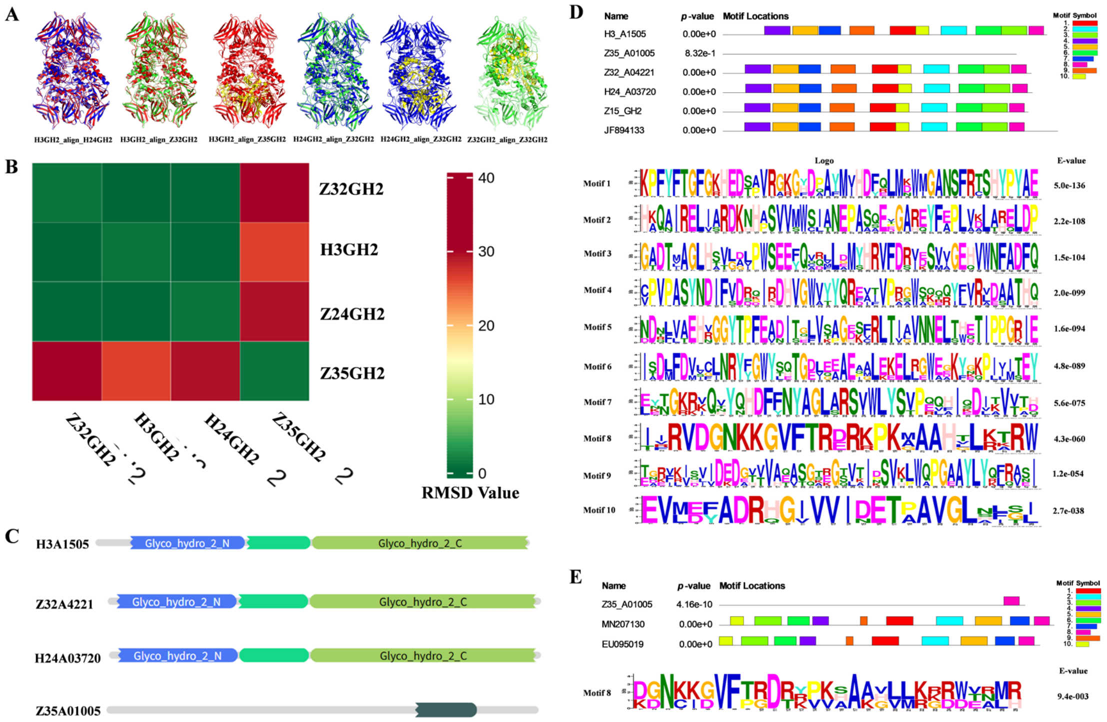

## Table S1. Primer sequences used for PCR amplification

| Gene ID | Primer sequence (5'——3') |
|---|---|
| H3A1505 | F: gccatggctgatatcggatccATGAGGTTCCTCACGGGATTG<br>R: acggagctcgaattcggatccTTACCCACTTGTGGTCATATTTGTC |
| Z9A4232 | F: gccatggctgatatcggatccATGTGGCAACTTCAAGGCTTTG<br>R: acggagctcgaattcggatccTTAGAAGAGACTCATCATAACGCTGG |
| Z9A0411 | F: gccatggctgatatcggatccATGGTCCACGACTATCGCTTG<br>R: acggagctcgaattcggatccCTAAGCTTGCTTTTTACCATTCTCTT |
| H16A06307 | F: gccatggctgatatcggatccATGCTCAAGCCTCAGGCAAA<br>R: acggagctcgaattcggatccCTAAGCTTGCTTTTTACCATTCTCTT |
| Z35A05952 | F: gccatggctgatatcggatccATGTTGCGACCCCAAGCA<br>R: acggagctcgaattcggatccTCAATCGCCGTGGGTTTG |
| Z35A10889 | F: gccatggctgatatcggatccATGCTCAAACCACAGGCCAA<br>R: acggagctcgaattcggatccTTAGGCCTGAACATTGCCTTTC |
| Z32A04221 | F: gccatggctgatatcggatccATGTTGCGACCCCAAGCA<br>R: acggagctcgaattcggatccTCAATCGCCGTGGGTTTG |
| Z32A8185 | F: gccatggctgatatcggatccATGCTCAAACCACAGGCCAA<br>R: acggagctcgaattcggatccTTAGGCCTGAACATTGCCTTTC |
| Z35A01005 | F: tcgagctccgtcgacaagcttATGGCTGGGACTGGGGTCC<br>R: ctcgagtgcggccgcaagcttTCATCCGACATTCGACACCC |
| Z11A0724 | F: gccatggctgatatcggatccATGCTCAAGCCTCAGGCAAA<br>R: acggagctcgaattcggatccCTAAGCTTGCTTTTTACCATTCTCTT |
| H24A3720 | F: gccatggctgatatcggatccATGCTCAAACCACAGGCCAA<br>R: acggagctcgaattcggatccTTAGACCTGAACATTGCCTTTCTTG |
| Z25A00123 | F: gccatggctgatatcggatccATGTCCGACGTGCAAGTTCAC<br>R: acggagctcgaattcggatccTCATAACCGTCTAAAGTTAGGGTTGC |
| H3A1505 | F: caggtcgactctagaggatccAGATGAGGTTCCTCACGGGATT<br>R: gagctcggtacccggggatccTTACCCACTTGTGGTCATATTTGTC |
| Z32A04221 | F: caggtcgactctagaggatccAGATGTTGCGACCCCAAGC<br>R: gagctcggtacccggggatccTCAATCGCCGTGGGTTTG |
| Z35A01005 | F: caggtcgactctagaggatccAGATGGCTGGGACTGGGGT<br>R: gagctcggtacccggggatccTCATCCGACATTCGACACCC |
| H24A3720 | F: caggtcgactctagaggatccATGCTCAAACCACAGGCCAA<br>R: gagctcggtacccggggatccTTAGACCTGAACATTGCCTTTCTTG |
| H3A1505 | qF: CCACTCATCACGGTCGTATC<br>qR: GGTAAGCTCGTTGTTGACAC |
| Z32A04221 | qF: CGAGCTTACCTGGGACACTA<br>qR: AGTATAGCCAGACGGAACGA |
| Z35A01005 | qF: CAGTACCCCTGTTACCCAGA<br>qR: GAGCAGTAGTTTCTCAGCC |
| Z24A3720 | qF: GAGGCTACACACCATTCGAG<br>qR: CTGCCAGGTGGTATTGTCTG |

| Gene ID | Primer sequence (5'——3') |
| --- | --- |
| Z32/35SSD | qF: CTGTCCATGACGACACATCC |
| | qR: CCTCGAAGACGATGAATGGG |
| H24SSD | qF: GCTGGTACGACCTGATGATG |
| | qR: CCGAGAACCATTCGAGGAAC |
| H3Actin | qF: AGAGCACCCCATTCTCTTGA |
| | qR: GGATGGAGACGTAGAATGCG |
| NbEF1-α | qF: AAGATGATTCCGACCAAGCC |
| | qR: CAACAGTTTGACGCATGTCC |
| ITS1 | TCCGTAGGTGAACCTGCGG |
| ITS4 | TCCTCC GCTTATTGATATGC |

**Table S2. Whole-genome assembly of nine licorice endophytic fungi.**

| Sample_name | Total Length(bp) | Gene Number （>500 bp） | N50 length （bp） | N90 length （bp） | Sequence GC % |
|---|---|---|---|---|---|
| H3 | 31,851,176 | 249 | 449,013 | 131,977 | 47.86 |
| H16 | 69,922,892 | 2,435 | 251,409 | 18,135 | 51.91 |
| H24 | 59,884,928 | 819 | 411,929 | 75,414 | 48.44 |
| Z9 | 58,371,146 | 2,383 | 264,349 | 15,926 | 49,24 |
| Z11 | 58,563,869 | 4,000 | 258,694 | 7,836 | 49.96 |
| Z22 | 58,624,796 | 3,228 | 288,264 | 9,629 | 49.94 |
| Z25 | 59,100,390 | 4,217 | 262,436 | 7,191 | 50.48 |
| Z32 | 56,068,852 | 1,075 | 397,369 | 45,475 | 50.04 |
| Z35 | 60,392,993 | 2,973 | 182,111 | 9,012 | 51.16 |

**Table S3. 3D structure prediction results for 12 GH2 candidate proteins**

| Gene ID | EMQE value | Sequence confidence (%) |
|---|---|---|
| H3A1505 | 0.86 | 71.86 |
| H16A06307 | 0.85 | 62.16 |
| Z35A05952 | 0.86 | 60.81 |
| Z35A10889 | 0.84 | 59.85 |
| Z32A04221 | 0.86 | 60.81 |
| Z32A8185 | 0.85 | 60.3 |
| Z11A0724 | 0.85 | 62.2 |
| H24A3720 | 0.85 | 60.91 |
| Z35A101005 | 0.93 | 70.5 |
| Z9A4232 | 0.96 | 94.53 |
| Z9A0411 | 0.81 | 65.74 |
| Z25A00123 | 0.95 | 91.08 |

**Table S4. Molecular docking scores of GH2 candidate proteins with glycyrrhizin ligands**

| Gene ID | Docked energy with GL (kcal/mol) |
|---|---|
| H3A1505 | -4.25 |
| H16A06307 | -5.33 |
| Z35A05952 | -7.05 |
| Z35A10889 | -5.44 |
| Z32A04221 | -3.45 |
| Z32A8185 | -3.6 |
| Z11A0724 | -5.63 |
| H24A3720 | -3.53 |
| Z35A101005 | -4.46 |
| Z9A4232 | -6.43 |
| Z9A0411 | -4.13 |
| Z25A00123 | -4.13 |

## Table S5. Conserved structural features and functional predictionsof GH2 proteins

| *β*-Glucuronidase | Structures | description | start | end | Biological Process | Molecular Function |
|---|---|---|---|---|---|---|
| H3GH2 | Glycosyl hydrolases family 2 | sugar binding domain | 52 | 219 | glucuronoside catabolic process (GO:0019391) | carbohydrate binding (GO:0030246); beta-glucuronidase activity (GO:0004566) |
| | Immunoglobulins | glycosyl hydrolases family 2 | 222 | 315 | | |
| | | beta-Galactosidase/glucuronidase domain | 412 | 494 | | |
| | Glycosyl hydrolases family 2 | TIM barrel domain | 317 | 633 | | |
| Z32GH2 | Glycosyl hydrolases family 2 | sugar binding domain | 13 | 180 | glucuronoside catabolic process (GO:0019391) | carbohydrate binding (GO:0030246); beta-glucuronidase activity (GO:0004566) |
| | Immunoglobulins | glycosyl hydrolases family 2 | 183 | 280 | | |
| | | beta-Galactosidase/glucuronidase domain | 183 | 281 | | |
| | Glycosyl hydrolases family 2 | TIM barrel domain | 282 | 596 | | |
| H24GH2 | Glycosyl hydrolases family 2 | sugar binding domain | 13 | 181 | glucuronoside catabolic process (GO:0019391) | carbohydrate binding (GO:0030246); beta-glucuronidase activity (GO:0004566) |
| | Immunoglobulins | glycosyl hydrolases family 2 | 183 | 281 | | |
| | | beta-Galactosidase/glucuronidase domain | 183 | 282 | | |
| | Glycosyl hydrolases family 2 | TIM barrel domain | 283 | 597 | | |
| Z35GH2 | - | glycosidases | 45 | 373 | glycoprotein catabolic process (GO:0006516) | beta-mannosidase activity (GO:0004567) |
| | | mannosidase Ig/CBM-like domain | 412 | 494 | | |
| | | beta-Galactosidase/glucuronidase domain | 509 | 572 | | |

**Table S6. Glycosyl hydrolase family 2 signatures**

| | H3GH2 | | Z32GH2 | | H24GH2 | |
|---|---|---|---|---|---|---|
| Signature | start | end | start | end | start | end |
| 1 | 326 | 340 | 291 | 305 | 292 | 306 |
| 2 | 357 | 375 | 322 | 340 | 323 | 341 |
| 3 | 440 | 455 | 406 | 421 | 406 | 421 |
| 4 | 539 | 554 | 505 | 520 | 505 | 520 |

**Table S7. Correlation between endogenous fungal glycyrrhizin hydrolyzing *GH2* gene expression levels and glycyrrhizin content in reinfected licorice roots**

| | *H3GH2* | *H24GH2* | *Z32GH2* | *Z35GH2-1* | *Z35GH2-2* |
|---|---|---|---|---|---|
| **GL content** | 0.806** | 0.122 | 0.885** | 0.786* | 0.476 |
| ***p*_value** | 0.009 | 0.754 | 0.002 | 0.012 | 0.195 |

"*", $p$<0.05, "**", $p$<0.01;
